# A Retrospective Evaluation of the Diagnostic Performance of an Interdependent Pairwise MicroRNA Expression Analysis with a Mutation Panel in Indeterminate Thyroid Nodules

**DOI:** 10.1089/thy.2022.0124

**Published:** 2022-11-11

**Authors:** Sydney D. Finkelstein, John W. Sistrunk, Carl Malchoff, Diane V. Thompson, Gyanendra Kumar, Venkata Arun Timmaraju, Brittany Repko, Alidad Mireskandari, Lisa Ann Evoy-Goodman, Nicole A. Massoll, Mark A. Lupo

**Affiliations:** ^1^ Interpace Diagnostics, Parsippany, New Jersey, USA.; ^2^ Jackson Thyroid and Endocrine Clinic, Jackson, Mississippi, USA.; ^3^ UConn Health, Neag Comprehensive Cancer Center, Farmington, Connecticut, USA.; ^4^ Diane Vido Thompson, LLC, Murrysville, Pennsylvania, USA.; ^5^ University of Arkansas for Medical Sciences, Little Rock, Arkansas, USA.; ^6^ Thyroid and Endocrine Center of Florida, Sarasota, Florida, USA.

**Keywords:** miRNA, expression profiling, molecular testing, thyroid

## Abstract

**Background:**

The addition of genetic analysis to the evaluation of thyroid nodule fine-needle aspiration biopsy samples improves diagnostic accuracy of cytologically indeterminate thyroid nodules (ITNs) with Bethesda III or IV cytopathology. We previously reported the performance of a multiplatform molecular test, referred to in this study as MPTXv1, that includes a mutation panel (ThyGeNEXT^®^) plus an algorithmic microRNA (miRNA) risk classifier (ThyraMIR^®^). Complex interactions of growth-promoting and -suppressing miRNAs affect the phenotype. We previously demonstrated that accounting for these interactions with pairwise miRNA expression analysis improves the diagnosis of medullary thyroid carcinoma. In this study, we assess the impact of pairwise miRNA expression analysis on risk stratification of ITNs.

**Methods:**

Pairwise expression analysis of 11 miRNAs was performed on a training cohort of histopathology-proven benign nodules (*n* = 50) to define the mean and standard deviation of each pairwise analysis and create a Benign/Malignant Profiler (MPTXv2), deviations from which predicted the malignancy risk. Clinical validation of MPTXv2 was assessed using a cohort of 178 ITN (Bethesda III and IV) samples from a multicentered, blinded retrospective study, previously evaluated by MPTXv1.

**Results:**

Compared with MPTXv1, MPTXv2 significantly improved the test performance. The receiver operating characteristic (ROC) areas under the curve (AUC) increased from 0.85 to 0.97 (*p* < 0.001), and the diagnostic accuracy at the positive threshold increased significantly (*p* < 0.05) from 83% [95% confidence interval (CI) = 76–88] to 93% [CI = 89–96]. The significant improvement in the ROC AUC and the diagnostic accuracy was due to a strong statistical trend for improvement in specificity at the positive threshold. At the positive threshold, the specificity for MPTXv1 was 90% [CI = 84–95] and improved to 98% [CI = 94–99] for MPTXv2. Using the MPTXv2, the Moderate-Risk cohort decreased from 50 samples (28% of the cohort) to 24 samples (13% of the cohort). This 52% decrease is statistically significant (*p* < 0.001) and clinically meaningful.

**Conclusion:**

As compared with MPTXv1, pairwise miRNA expression analysis used in MPTXv2 significantly improved the diagnostic accuracy of ITN risk stratification and reduced the size of the Moderate-Risk group. Prospective trials are indicated to confirm these findings in a clinical practice setting.

## Introduction

Phenotypic expression of thyroid neoplasia, whether differentiating between benign versus malignant status or identifying specific pathologic entities reflecting abnormal cell proliferation, involves the combination of genomic and epigenomic factors.^1,2^ Epigenomic growth regulation includes a variety of cellular mechanisms, including differential microRNA (miRNA) expression that controls protein translation.^3,4^ Strong-driver, BRAF V600E-like mutations, are highly predictive of malignancy but occur in extremely low frequency in benign nodules.^5,6^ Weak-driver, *RAS*-like mutations are common but much less predictive of malignancy.^7,8^ Additional epigenomic factors, including miRNAs, likely contribute to the ultimate phenotype.

In particular, two diagnostic challenges are those indeterminate thyroid nodules (ITNs) with Bethesda III and Bethesda IV cytopathology results that have no identifiable mutation or carry activating *RAS* mutations, and those ITN that are Hürthle cell-predominant (HCP). A *RAS* mutation has limited predictive value, since these mutations are found frequently in both benign follicular adenomas (FA) as well as a variety of thyroid malignancies.^7–17^ An isolated *RAS*-like mutation requires further information to accurately predict malignancy risk.^11,12^ It is not possible to discriminate benign from malignant HCP nodules by cytology alone.^13,14^

We recently reported the analytical validation^18^ and clinical validation^19^ of an expanded mutation panel in combination with a miRNA risk classifier for diagnosis of ITNs. This clinical validation study employed a single computational algorithm based on the independent expression levels of 10 specific miRNAs.^20,21^ This multiplatform molecular testing (MPTX, referred to as MPTXv1 in this article) delivers malignancy risk in three result levels: Negative (low risk), Moderate-Risk, or Positive (high risk). Epigenetic growth regulators are likely interdependent. For example, we previously reported that pairwise miRNA expression profiling methodology accurately identifies medullary thyroid carcinoma.^22^

We hypothesize that the malignancy risk of both *RAS*-mutated and HCP ITNs can be refined by evaluating the interaction of miRNA expression changes. In the current study, we apply pairwise miRNA expression analysis to ITN, with the goals to: 1) further increase the Negative Predictive Value (NPV) and Positive Predictive Value (PPV) of MPTX; 2) to reduce the proportion of samples assigned to the Moderate-Risk category; and 3) improve risk stratification of nodules with *RAS*-like mutations and those containing Hürthle cells.

## Materials and Methods

### Fine needle aspirate samples from subjects

The study cohort included two separate ITN cohorts (a training cohort and a clinical validation cohort). An independent Ethics Review Board (Advarra IRB #33697) approved this study and waived informed consent.

The training cohort consisted of 50 cytologically ITN (Bethesda III and IV) that were confirmed to be benign by final surgical histopathology. There were 24 hyperplastic nodules, 20 FA, and 6 Hürthle cell adenomas (HCA).

The previously described clinical validation cohort consisted of 197 ITN (Bethesda III, IV, or V) samples from a fully blinded, multicenter, retrospective study employed for clinical validation of MPTXv1. In brief, from an initial identification of 309 cytologically ITNs, 57 were excluded due to inadequate DNA for analysis. Of the remaining 252 ITN, 9 were excluded due to discordance between nodule excised and nodule biopsied or insufficient histopathology slide, and 46 were excluded due to failure of 3 independent pathologists to agree on final histopathology of the Hematoxylin and Eosin-stained tissue. Of the 197 ITNs, 92 were Bethesda III, 86 were Bethesda IV, and 19 were Bethesda V. Cytologic Hürthle cell change was recognized in 64. On final surgical pathology, 127 were benign and 70 were malignant (including noninvasive follicular thyroid neoplasm with papillary-like nuclear features [NIFTP]).^19^

### Molecular testing

The DNA mutational analysis and miRNA expression results were performed in the commercially available (Interpace Diagnostics) CLIA and CAP-accredited laboratory in Pittsburgh, PA. In the commercial assay, all samples first undergo testing with the mutation panel (ThyGeNEXT). When a strong-driver (*BRAF*-like mutation) is found, miRNA analysis is not required and is not performed. Only samples with a weak driver or no mutation undergo miRNA analysis. In contrast, in this study, all samples underwent both the algorithmic and the pairwise miRNA analysis, including those that had a strong driver, BRAF-like mutation identified. The performance of the miRNA pairwise analysis was blinded to the histopathology and cytopathology results of the validation cohort. Molecular test results were stored in a secure laboratory information system database that was password protected and separate from the database that harbored deidentified baseline characteristics and follow-up histopathology diagnoses of subjects.

### Development of the benign/malignant miRNA expression profiler

Using a training set of 50 ITN (Bethesda III and IV) with final benign histopathology, the cycle threshold (Ct) was determined for the 6 growth-promoting miRNAs (miR-21, -31, -146, -222, -375, -551) and for the 5 growth-suppressing miRNAs (miR-29, -138, -139, -155, -204) using quantitative RT-PCR (Quant Studio) as described previously.^19^ This was followed by pairwise analysis of the Ct values. Cohort size was judged adequate with there being no further improvement in mean and standard deviation (SD) or each pairwise measurement.

For the validation cohort, the miRNA risk classifier (ThyraMIR) using quantitative RT-PCR (Quant Studio) to determine the Ct of 10 miRNAs and the algorithmic classifier analysis were described previously.^19^ In addition, in the current study, the Ct of thyroid growth-promoting miRNA, miR-21, was determined for all of the samples. Pairwise analysis was applied to the Ct values of these 11 miRNAs (10 miRNA marker panel, plus miR-21).

**FIG. 1.**
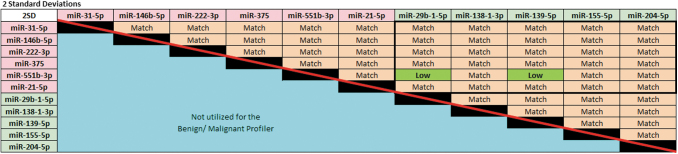
Pairwise analysis for benign versus malignant discrimination. The balance between growth promotion (six miRNAs) and growth suppression (five miRNAs) reflects the overall equilibrium in growth status (bold outlined box). Averages for each pair were calculated using a known dataset (*n* = 50) of histologically confirmed benign thyroid nodules. Values falling within two SDs are labeled as a match with two pairs labeled as low, which also favor growth suppression or a benign diagnosis. These miRNAs are all established markers in thyroid cancer biology. miRNA, microRNA; SDs, standard deviations.

A growth balance value was calculated for each sample by canceling out promotion versus suppression and then analyzing the remainder values. Scoring was based on one and two SDs assigning a single point for each pairwise result falling beyond the SD. Values exceeding two SDs would have a higher score and be reflective of greater imbalance. Our analysis showed that 30 pairwise relationships exist between individual growth-promoting and growth-suppressing miRNA pairs (Fig. 1).

The Benign/Malignant Profiler (MPTXv2) classification criteria were selected to meet predetermined statistical frequencies of a normal distribution: (1) approximately 84% Negative-final total showing three or more growth-suppressing pairs; (2) 13.5% Moderate-Risk-total showing two or fewer growth-suppressing pairs or up to five growth-promoting pairs; and (3) 2.5% Positive-total showing six or more growth-promoting pairs. In the final classification, all samples with strong-driver, BRAF V600E-like mutations were classified as Positive, regardless of the miRNA status. For the samples with no mutation or a *RAS*-like mutation, the MPTXv2 took precedence over MPTXv1.

### Statistical analysis

Diagnostic sensitivity, specificity, NPV, PPV, and accuracy of MPTXv1 were compared with that of MPTXv2 that includes pairwise miRNA expression analysis. Receiver Operating Characteristic (ROC) Curve Analysis was used to assess the overall quality of two diagnostic tests with 3-category results, comparing the MPTXv1 stratification methodology^19,23^ with MPTXv2 using the entire study cohort (B-III, B-IV, and B-V; *n* = 197) and a subset (with only B-III, and B-IV samples (*n* = 178). Areas Under the Curve (AUC) were computed empirically, without parameterization or smoothing. Standard Errors were computed as per DeLong et al,^24^ 95% confidence intervals (CIs) for AUC's were computed using the binomial exact method. The diagnostic tests were then compared against each other by checking the *difference* in AUCs for statistical significance, with a value of *p* < 0.05 indicating that statistically, the test with the higher AUC was a significantly better predictor. CIs for Sensitivity and Specificity were computed as exact Clopper–Pearson CIs. Standard performance characteristics were calculated for a Positive result alone (positive threshold), or for a Moderate result or Positive result (moderate threshold).

PPV and NPV were calculated at the positive and moderate thresholds, respectively. CIs for PPV and NPV are the standard logit CIs given by Mercaldo et al.^25^ McNemar's test for paired proportions was used to determine if the proportion of total samples classified as Moderate-Risk was different in the MPTXv2 as compared with the MPTXv1 and if the proportion of *RAS*-mutated samples classified as Moderate-Risk was different in the MPTXv2 as compared with MPTXv1.

Data were analyzed using MedCalc Software, version 20.011 (MedCalc Software Ltd., Ostend, Belgium). Separate analyses were carried out for all samples B-III, B-IV, and B-V; (*n* = 197), and for the subset of only B-III and B-IV samples (*n* = 178). An additional Supplementary analysis was performed with the Moderate-Risk test results added to the Positive test results to calculate binary performance metrics (Supplementary Table S1); in this analysis, NIFTP samples were scored as malignant and disease prevalence of 30% was used to compute PPV and NPV.

## Results

### Overall performance of MPTXv2 compared with MPTXv1

**FIG. 2.**
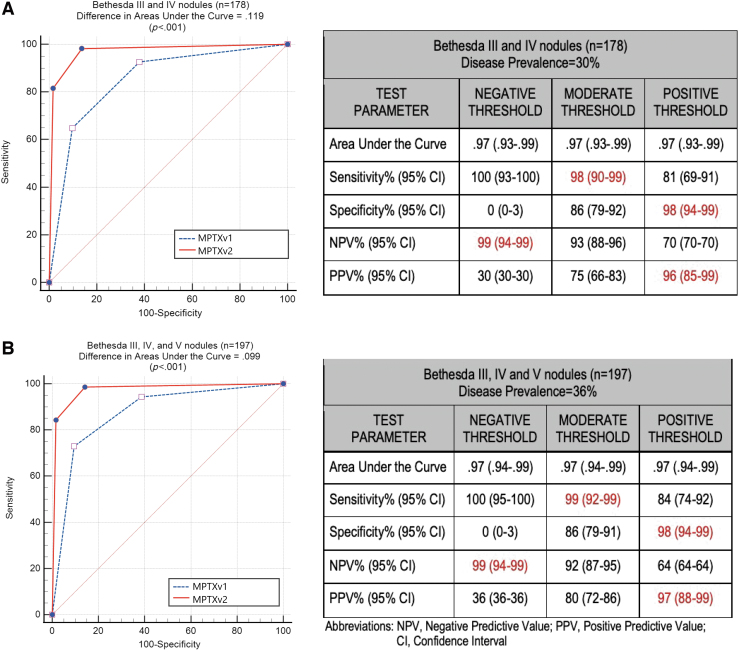
ROC curve comparisons between MPTXv1 and MPTXv2 in a subgroup of Bethesda III and IV nodules (n-178) with assumed ROD = 30%, as shown in the upper half of the figure **(A)**, and for the entire cohort of Bethesda III, IV, and V nodules (*n* = 197) with assumed ROD = 36%, as shown in the lower half of the figure **(B)**. Tables on the right contain test performance metrics for MPTXv2. ROC, receiver operator characteristics; ROD, rate of disease.

The MPTXv2 substitutes the Benign/Malignant pairwise miRNA analysis for the algorithmic miRNA analysis used in MPTXv1. Table 1 shows the distribution of test calls relative to the final histopathology diagnosis for the MPTXv1 and MPTXv2 analyses in the B-III/B-IV subgroup (Table 1a), the entire clinical validation cohort (B-III/B-IV/B-V; Table 1b), and separated into the B-III, B-IV, and B-V subgroups (Table 1c). The ROC curves (Fig. 2) describe the three-tier risk stratification (Negative, Moderate-Risk, Positive) and compare the previously reported MPTXv1 test results^19^ with those of the MPTXv2. As compared with MPTXv1, the MPTXv2 analysis significantly improved the ROC AUC for both the B-III/B-IV clinical validation subset (*n* = 178) and the entire clinical validation cohort (B-III/B-IV/B-V; *n* = 197) (Difference in AUC of .119 and .099, respectively, *p* < 0.001 for both). The diagnostic accuracy at the positive threshold increased significantly (*p* < 0.05) from 83% [CI = 76–88] for the MPTXv1 to 93% [CI = 89–96] for MPTXv2.

**Table 1.** Distribution of Test Results (MPTXv1 vs. MPTXv2) for Samples with Unanimous Histological Diagnosis

(a) Samples with B-III and B-IV cytology (*n* = 178)				
	Benign	Malignant+NIFTP		Total
MPTXv1				
Negative	77	4		81
Moderate	35	15		50
Positive	12	35		47
Total	124	54		178
MPTXv2				
Negative	107	1		108
Moderate	15	9		24
Positive	2	44		46
Total	124	54		178

(b) Samples with B-III, B-IV, and B-V cytology (*n* = 197)				
	Benign	Malignant+NIFTP		Total
MPTXv1				
Negative	78	4		82
Moderate	37	15		52
Positive	12	51		63
Total	127	70		197
MPTXv2				
Negative	109	1		110
Moderate	16	10		26
Positive	2	59		61
Total	127	70		197

(c) Comparison of change in MPTXv1 and MPTXv2 calls based on cytology category				
	Neg	Mod	Pos	Total
Bethesda | MPTXv1				
B-III	38	27	27	92
B-IV	43	23	20	86
B-V	1	2	16	19
B-III, B-IV	81	50	47	178
B-III, B-IV, B-V	82	52	63	197
Bethesda | MPTXv2				
B-III	53	11	28	92
B-IV	55	13	18	86
B-V	2	2	15	19
B-III, B-IV	108	24	46	178
B-III, B-IV, B-V	110	26	61	197

(a) Sample distribution for MPTXv1 and MPTXv2 results for samples with B-III and B-IV cytology and unanimous histological diagnosis (*n* = 178). (b) Overview of sample distribution for MPTXv1 and MPTXv2 results for samples with B-III, B-IV, and B-V cytology and unanimous histological diagnosis (*n* = 197). MPTXv2 reduced false positives, false negatives, and the number of samples in the Moderate-Risk group. (c) Comparison of change in MPTXv1 and MPTXv2 calls based on cytology category.

The significant improvement in the ROC AUC and the diagnostic accuracy was due to a strong statistical trend for improvement in specificity at the positive threshold plus a modest improvement in sensitivity (Fig. 2). For the B-III/B-IV subset (*n* = 178) at the positive threshold, the specificity was 90% [CI = 84–95] for MPTXv1^19^ and improved to 98% [CI = 94–99] for MPTXv2. Similar results were obtained for the B-III/B-IV/B-V cohort (*n* = 197).

A secondary two-tiered analysis in which the Moderate-Risk samples were classified as Positive for malignancy is shown in Supplementary Table S1. As compared with MPTXv1,^19^ the MPTXv2 significantly improved the specificity and test accuracy for the B-III/B-IV subgroup. The specificity increased from 62% [CI = 53–71] to 86% [CI = 79–92], and the diagnostic accuracy increased from 71% [CI = 64–78] to 90% [CI = 84–94]. This resulted in a significant increase in the PPV from 51% [CI: 45–57] to 75% [CI = 66–83]. Not shown are similar results for the entire cohort.

There were clinically important differences between the MPTXv1 and MPTXv2 results. Table 1 shows the reclassification of the MPTXv1 Moderate-Risk samples by the MPTXv2. Overall, there was a 52% reduction (*p* < 0.001) in the number of B-III/B-IV subjects in the Moderate-Risk category, from 50 to 24. MPTXv2 reclassified with 100% accuracy 34 of the MPTXv1 Moderate-Risk samples. Ten were reclassified as true positives and 24 were reclassified as true negatives (Table 2). There were eight samples in the entire cohort (B-III, B-IV, and B-V) originally called Positive by MPTXv1 that were reclassified as Moderate-Risk by MPTXv2 (two FA, 1 HCA, three papillary thyroid carcinomas [PTC], and two NIFTP). Of the three PTCs, two had no mutations and one had a *RAS*-like mutation. Of those samples with a *RAS*-like mutation, the number classified as Moderate-Risk decreased significantly (*p* < 0.001) from 39 with the MPTXv1 to 16 with the MPTXv2.

**Table 2.** Sample Characteristics for Moderate-Risk Samples with No Mutation or a *RAS*-Like Mutation Along with Corresponding MPTXv1 and MPTXv2 Test Results

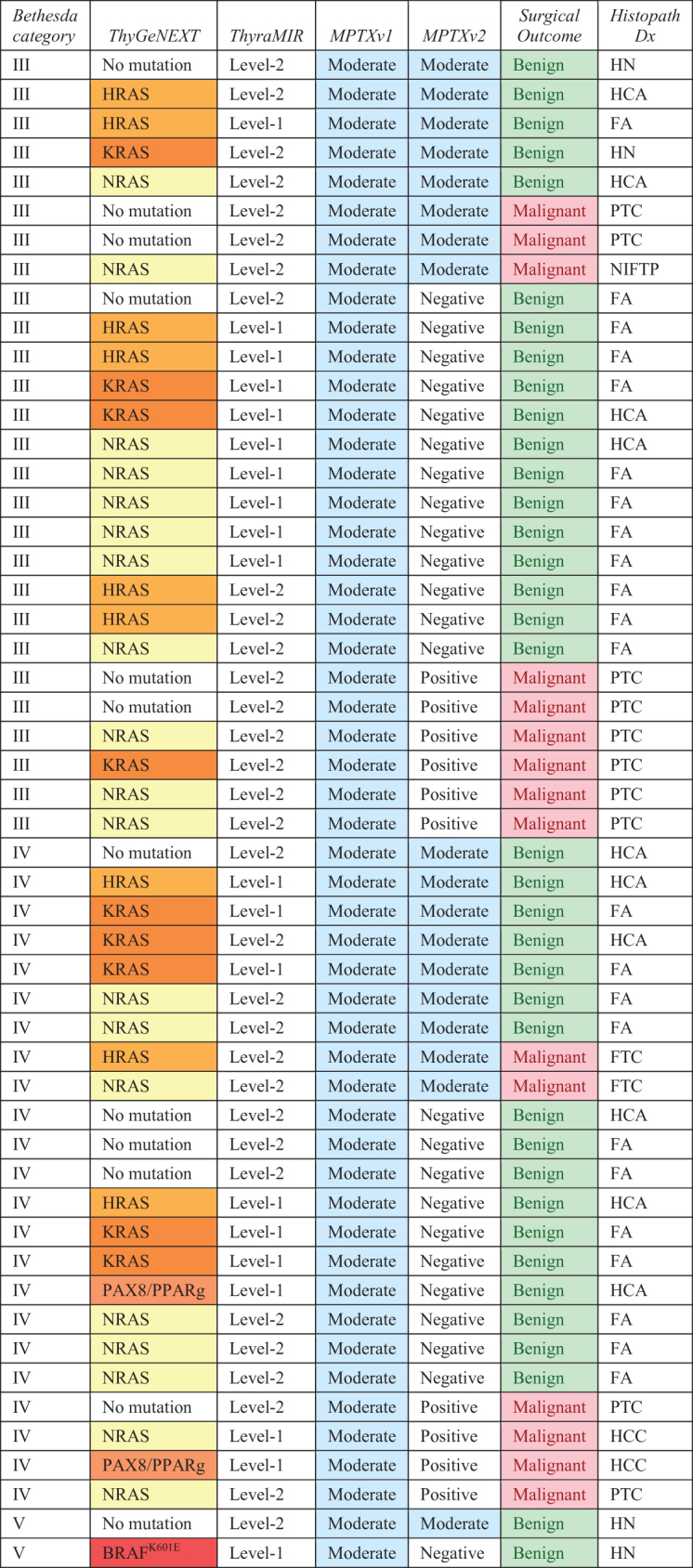

Overview of samples with no mutations or *RAS*-like mutations (*n* = 52) that were assigned to the Moderate-Risk group by MPTXv1 (no mutation, *n* = 12; *RAS*-like mutation, *n* = 40). With 100% accuracy, MPTXv2 correctly reclassified the majority of samples, with *RAS*-like mutations or no mutation that were originally in the MPTXv1 Moderate-Risk group. It also highlights the improved performance for Hürthle cell lesions that were part of the Moderate-Risk group.

FA, follicular adenomas; FTC, follicular thyroid carcinoma; HCA, Hürthle cell adenomas; HN, hyperplastic nodules; PTC, papillary thyroid carcinomas.

**FIG. 3.**
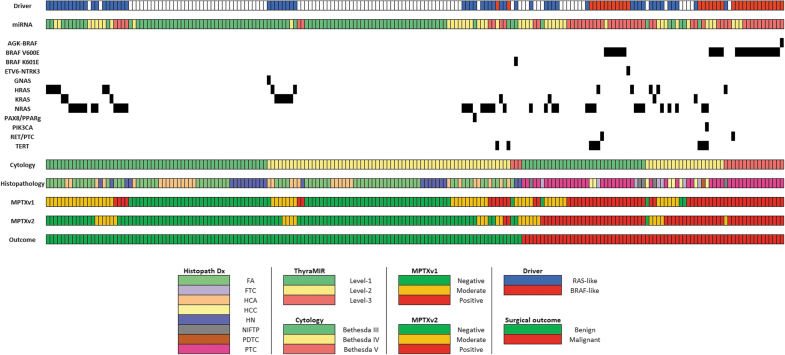
Distribution of samples by cytological diagnosis, histopathology diagnosis, and molecular testing results. Novel miRNA pairwise expression profiler (MPTXv2) applied to a cohort of 197 samples with known histology (each column represents a single patient case). MPTXv2 is effective across the different genotypes of weak-driver mutations dominated by *RAS-*like gene mutations. MPTXv2 accurately reclassified the majority of the samples that were Moderate-Risk by MPTXv1.

The MPTXv2 accurately reclassified three of the four previous false negatives (Table 1); two patients had minimally invasive follicular carcinomas and one had a follicular variant of PTC (Fig. 3). The one false negative was a Hürthle cell cancer.

The ROD (Rate of Disease) for the MPTXv1 Moderate-Risk cohort was observed to be 30% and was 28% for samples that remained in the Moderate-Risk group following MPTXv2 analysis. There was no correlation observed in the genotype of the samples that were correctly reclassified by MPTXv2.

### Benign/malignant profiling in relation to microscopic and molecular features

The Moderate-Risk MPTXv1 samples, with no mutations or a *RAS*-like mutation that were recategorized by MPTXv2, were all accurately reclassified by MPTXv2 (Table 2, Fig. 3). Improved risk classification was seen across all three *RAS* gene families. Of 37 B-III/B-IV *RAS*-mutated nodules originally assigned to the Moderate-Risk category, only 13 samples remained in the Moderate-Risk category after the Benign/Malignant Profiler was applied (10 benign, 3 malignant) (*p* < 0.001). The use of the Benign/Malignant Profiler increased the diagnostic accuracy for nodules with *RAS*-like mutations by a substantial reduction in false positives and in the number of samples in the Moderate-Risk group (Table 2, Fig. 3).

Table 1 shows that the number of Negative results in the entire cohort increased significantly from 78 to 109 (*p* < 0.001). The majority of the reclassified benign nodules were FA, which increased the number of Negative results by 26. Reclassification of samples with nodular hyperplasia increased from 18 to 21 nodules. The Benign/Malignant Profiler (MPTXv2) accurately ruled-in malignancy in 10 nodules (2 Hürthle cell carcinomas [HCC] and 8 PTC) that were previously categorized as Negative or Moderate-Risk by MPTX alone (Fig. 3).

### HCP nodules

Hürthle cell change was recognized in 64/197 (33%) of samples: 22/70 (31%) of FA, 33/33 (100%) of HCA, 8/8 (100%) of HCC, and 1/49 (2%) of PTC. The risk of malignancy for HCP nodules classified as Moderate-Risk was low, both when assessed by MPTXv1 (13%) and for MPTXv2 (8%). The use of MPTXv2 correctly assigned two HCC samples as malignant that were previously called Moderate-Risk by MPTXv1, but this difference was not statistically significant (*p* = 0.58).

### Likelihood ratios and predictive values

The MPTXv2 likelihood ratios are shown in Table 3a for the B-III/B-IV cohort and in Table 3b for the entire B-III/B-IV/B-V cohort. There is clear separation of the CI of these three groups. In the setting of a known pretest probability, the likelihood ratio was used to calculate the post-test probability (predictive values). For the B-III/B-IV subset with a disease prevalence of 30%, the MPTXv1 PPV at positive threshold (post-test disease probability of a Positive result) was 74% [CI = 60–86]^19^ and increased to 96% [CI = 85–99] with the application of MPTXv2, a strong trend for improvement. Similar results were obtained for the entire cohort (*n* = 197). For the B-III/B-IV subset with a disease prevalence of 30%, the NPV (1 post-test disease probability of a Negative result) was 95% [CI = 88–99] using MPTXv1^19^ and increased slightly (*p* = NS) with the use of MPTXv2 to 99% [CI = 94–99]. Similar results were obtained for the entire cohort (*n* = 197). For the MPTXv2, 87% of all samples had a result that was either Negative or Positive for malignancy, with only 13% remaining in the Moderate-Risk group with a post-test malignancy risk of 38% [CI = 22–56].

**Table 3.** Likelihood Ratios

Risk	Cancer-positive	Cancer-negative	Likelihood ratio	CI
(a) MPTXv2 likelihood ratios for subjects with B-III and B-IV cytology (*n* = 178)				
Negative	1	107	0.0215	0.00307–0.150
Moderate	9	15	1.38	0.643–2.95
Positive	44	2	50.5	12.7–201
(b) MPTXv2 likelihood ratios for subjects with B-III, B-IV, and B-V cytology (*n* = 197)				
Negative	1	109	0.0166	0.00237–0.117
Moderate	10	16	1.13	0.544–2.36
Positive	59	2	53.5	13.5–212

The MPTXv2 likelihood ratios and their CI are shown for the B-III/B-IV cohort (*n* = 178) and for the entire B-III/B-IV/B-V cohort (*n* = 197).

CI, 95% confidence interval.

**FIG. 4.**
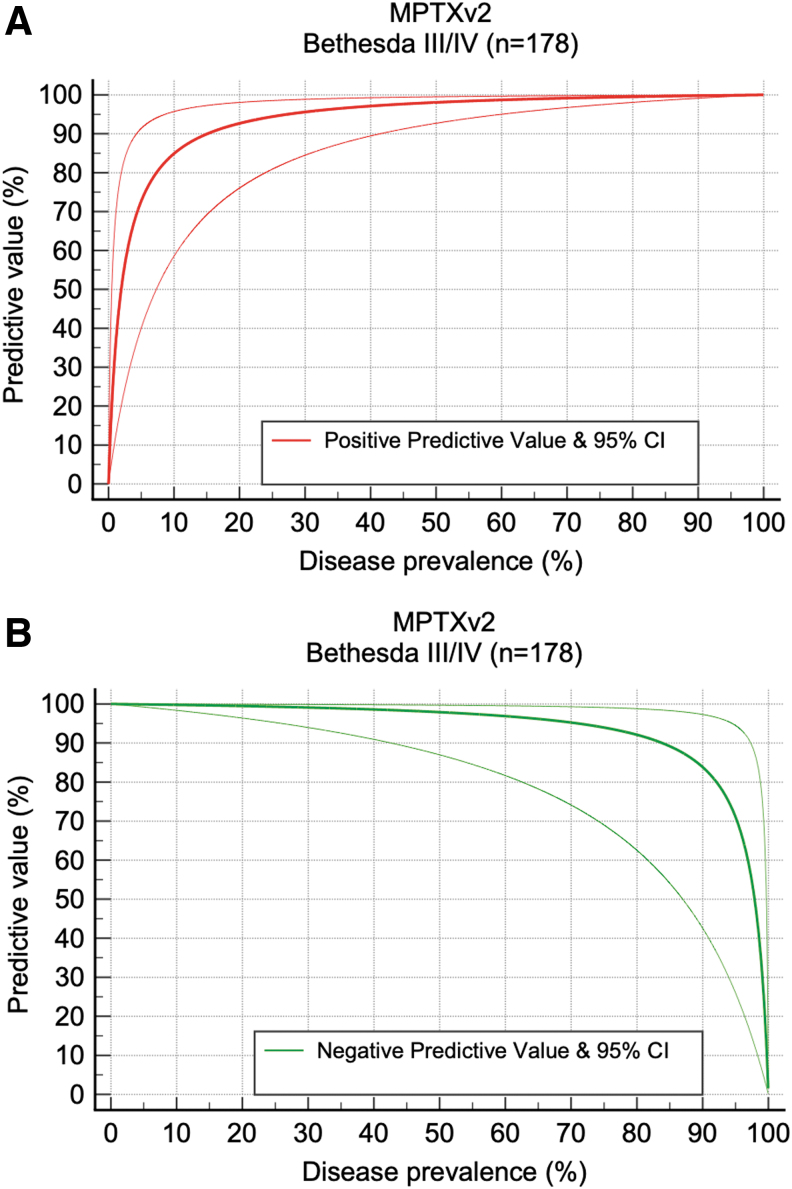
Effect of disease prevalence on PPV and NPV for MPTXv2 (B-III/B-IV; *n* = 178). **(A)** The effect of disease prevalence on the PPV (±2 SD) of a MPTXv2-positive result is shown in the red line. The expected post-test probability for a patient with a positive MPTXv2 result exceeds 90% at a disease prevalence above 16%. **(B)** The effect of disease prevalence on the NPV (±2 SD) of a MPTXv2-negative result is shown in the green line. The expected post-test probability for a patient with a Negative MPTXV2 result exceeds 90% at a disease prevalence below 84%.

Using the B-III/B-IV cohort (*n* = 178), Figure 4A shows the anticipated relationship of disease prevalence and PPV (post-test probability of a Positive result). Similarly, Figure 4B shows the anticipated relationship of disease prevalence and NPV of a Negative result (1 post-test disease probability of a Negative result). For very wide disease prevalence (16%–84%), the PPV of a Positive MPTXv2 result and the NPV of a Negative MPTXv2 result both exceed 90%.

## Discussion

Mutational analysis alone is often insufficient to accurately “rule-in” or “rule-out” malignancy in ITNs.^7–17^ We previously reported that the addition of quantitative analysis of miRNAs with an established role in thyroid carcinoma (ThyraMIR)^26,27^ to mutational analysis (ThyGeNEXT) improves the risk stratification of ITNs.^19,24^ This combined test is referred to in this study as MPTXv1. The miRNA analysis in this prior study utilized an algorithm based on miRNA expression derived by quantitative polymerase chain reaction of the 10 miRNA marker panel as previously validated^24,28^ to determine malignancy risk.^19^ In this current study, we incorporated a second measure of epigenomic control represented by the pairwise analysis of individual growth-promoting and growth-suppressing miRNAs. This new analysis (MPTXv2) provides clinically and statistically superior risk stratification.

Using the MPTXv2, a Positive or Negative result was provided in 154 out of 178 (86%) ITNs, while in the previous analysis (MPTXv1), a Positive or Negative result was provided in 128 out of 178 (52%). This improvement is largely due to an increase in the number of true negative results and reduction on false-positive results, with a subsequent improvement in the specificity and PPV. The Benign/Malignant Profiler (MPTXv2) was particularly effective in estimating the malignant potential of ITN with no mutation or with *RAS*-like mutations.

For the B-III/B-IV cohort at 30% disease prevalence, the malignancy risks of Positive, Moderate-Risk, and Negative samples are 99%, 38%, and 1%, respectively. Although 13% of samples remain in the Moderate-Risk category, it is preferable to report these as Moderate-Risk as opposed to combining them into the Positive category, as we did in our secondary analysis. The Moderate-Risk group is a separate group, since the CI of its likelihood ratios do not overlap with that of either the Positive or Negative group. Furthermore, this more precise risk estimation is very useful information for the clinician, who must integrate various risks and benefits when deciding against or for surgery and the extent of such surgery. The MPTXv2 compares very favorably with other available molecular tests for the malignant potential of ITN.^28,29^ However, other assays are also continuing to evolve and head-to-head studies, utilizing samples from the same nodule, are required to precisely compare performance. Since the disease prevalence in this study was 30%, a clinical practice outcome study with different disease prevalence may yield different PPV and NPV metrics.

We had predicted that miRNA analysis would predict malignancy risk in fine needle aspirate samples of ITN. Epigenomic changes have been well described in thyroid neoplasms^3,4^ emphasizing the need to look beyond DNA mutations/rearrangements and quantitative mRNA expression to more comprehensively account for and predict tumor biology. Since benign nodular disease is confined to two closely similar entities, hyperplasia and adenoma, it is reasonable that a single epigenomic miRNA-based algorithm may perform well to characterize benign states. This accounts for the high NPV (>95%) previously reported with MPTXv1.^19^

However, the diverse nature of malignant disease may not be predicted by an algorithm evaluating individual miRNAs. Since miRNAs alter gene expression at the level of mRNA translation into the proteins that promote and inhibit cell growth, then there is a potential for complex interactions between the multiple different miRNAs on any given mRNA translation and on different mRNAs. We hypothesized that a deeper analysis of miRNA expression by targeting the pairwise equilibrium between growth-promoting and growth-suppressing miRNAs would improve the prediction of malignancy risk.

We have already demonstrated the utility of pairwise analysis in the diagnosis of medullary thyroid cancer.^20^ As we had hypothesized, the MPTXv2, which explores miRNA interactions, provides more information about the malignant potential of thyroid nodules than MPTXv1. It also should be noted that miRNA analysis may reduce the risk of RNA sampling error. The analysis or miRNAs may have other advantages over mutational analysis and mRNA expression profiles. Since miRNAs can migrate throughout the nodule, they may be less affected by topographic heterogeneity than the distribution of cells with DNA mutations or benign/malignant mRNA expression profiles.

There are strengths and weaknesses of this study. This represents a clinical validation study, since MPTXv2 was evaluated in a fully blinded manner in a large cohort of nodules from several centers with unanimous consensus histopathology diagnosis. Weaknesses of this study include the retrospective design, the relatively large number of samples (18%) that were excluded due to failure of the three pathologists to agree on the final histopathology, and the relatively small number of samples for some histopathological subsets. This clinical validation cohort has been previously analyzed. Although a new clinical validation cohort would strengthen the study, the advent of molecular diagnosis has limited and skewed the availability of benign samples with final surgical histopathology, as was available in this cohort. As previously noted, 13% of samples remained in the MTXv2 Moderate-Risk category that provided no significant improvement in post-test probability. Samples were extracted from diagnostic cytology slides and did not include fresh aspiration material collected in preservative buffer. Finally, prospective trials are indicated to confirm these findings in a clinical practice setting.

In summary, MPTXv2 significantly increased the diagnostic accuracy of risk stratification of ITN and reduced the number of Moderate-Risk samples. We conclude that the addition of pairwise miRNA expression profiling analysis more accurately stratifies the malignancy risk of ITNs.

## Supplementary Material

Supplemental data
