# Supplementary material for: A Retrospective Evaluation of the Diagnostic Performance of an Interdependent Pairwise MicroRNA Expression Analysis with a Mutation Panel in Indeterminate Thyroid Nodules
Source: Thyroid. 2022 Nov 11;32(11):1362–71. doi: 10.1089/thy.2022.0124 (PMC9700378; doi:10.1089/thy.2022.0124)
Supplement: Supplemental data [file Suppl_TableS1.docx]

**Supplemental Table 1**. Binary test performance metrics for MPTXv1 versus MPTXv2 (B-III + B-IV cohort, n=178), with all Moderate-Risk and Positive test results being combined together and scored as Malignant/NIFTP. Highlighted in yellow are the specificity, PPV and test accuracy; the three performance metrics for which the MPTXv2 is superior to MPTXv1.

**Supplemental Table 1: Binary test performance metrics for B-III and B-IV nodules (n=178)**

| **Test Metric** | **MPTXv1 (95% CI)** | **MPTXv2 (95% CI)** |
| --- | --- | --- |
| Sensitivity | 93% (82 to 98%) | 98% (90 to 99%) |
| Specificity | 62% (53 to 71%) | 86% (79 to 92%) |
| AUC | 0.77 (0.70 to 0.83) | 0.92 (0.87 to 0.95) |
| Positive Likelihood Ratio | 2.44 (1.92 to 3.09) | 7.15 (4.59 to 11.15) |
| Negative Likelihood Ratio | 0.11 (0.04 to 0.30) | 0.02 (0.00 to 0.15) |
| Disease Prevalence | 30.00% | 30.00% |
| Positive Predictive Value | 51% (45 to 57%) | 75% (66% to 83%) |
| Negative Predictive Value | 95% (88 to 98%) | 99% (94% to 100%) |
| Accuracy | 71% (64 to 78%) | 90% (84% to 94%) |
